# Prevalence of *Yersinia enterocolitica* and *Yersinia pseudotuberculosis* in wild boars in the Basque Country, northern Spain

**DOI:** 10.1186/s13028-016-0184-9

**Published:** 2016-01-20

**Authors:** Maialen Arrausi-Subiza, Xeider Gerrikagoitia, Vega Alvarez, Jose Carlos Ibabe, Marta Barral

**Affiliations:** Department of Animal Health, Basque Institute for Agricultural Research and Development-NEIKER, Berreaga 1, 48160 Derio-Bizkaia, Spain

**Keywords:** *Yersinia enterocolitica*, *Yersinia pseudotuberculosis*, Wild boar, Epidemiology, PCR, ELISA

## Abstract

**Background:**

Yersiniosis is a zoonosis widely distributed in Europe and swine carry different serotypes of *Yersinia enterocolitica* and *Y. pseudotuberculosis*. The aim of this study was to determine the prevalence of *Y. enterocolitica* and *Y. pseudotuberculosis* in wild boars in northern Spain. The blood of wild boars (n = 505) was sampled between 2001 and 2012. Seroprevalence was determined in 490 serum samples with an indirect enzyme-linked immunosorbent assay. Seventy-two of the animals were also examined for the presence of *Y. enterocolitica* or *Y. pseudotuberculosis* in the tonsils with real-time polymerase chain reaction. All the tonsils were analysed twice, directly and after cold enrichment in phosphate-buffered saline supplemented with 1 % mannitol and 0.15 % bile salts.

**Results:**

Antibodies directed against *Y. enterocolitica* and *Y. pseudotuberculosis* were detected in 52.5 % of the animals. *Yersinia enterocolitica* was detected with real-time polymerase chain reaction in 33.3 % of the wild boars and *Y. pseudotuberculosis* in 25 %. Significant differences were observed according to the sampling year, and the highest prevalence was during winter and spring. The highest antibody levels and *Y.* *enterocolitica* prevalence were observed in mountainous areas at altitudes higher than 600 m, with very cold winters, and with the highest annual rainfall for each dominant climate. Areas with low and medium livestock populations were associated with the highest seroprevalence of *Yersinia* spp. in wild boars, whereas areas with high ovine populations had the highest prevalence of *Y. enterocolitica*.

**Conclusions:**

This study shows that *Y. enterocolitica* and *Y. pseudotuberculosis* are highly prevalent among wild boars in the Basque country, with *Y. enterocolitica* most prevalent. The risk of infection among wild boars is influenced by the season and the area in which they live.

**Electronic supplementary material:**

The online version of this article (doi:10.1186/s13028-016-0184-9) contains supplementary material, which is available to authorized users.

## Background

Yersiniosis is the fourth most frequently reported food-borne zoonosis in humans in Europe, although the number of reported cases of *Yersinia* infection has continued to decrease since 2007 [1]. The genus *Yersinia* is composed of several species, but only *Y. pestis*, *Y. pseudotuberculosis* and some *Y. enterocolitica* strains are human pathogens [1].

Pigs are assumed to be the main reservoir of human pathogenic *Y. enterocolitica*, and serotypes isolated from pig samples, such as 4/O:3, are the same that cause human disease in Europe [1]. *Yersinia pseudotuberculosis* has also been frequently isolated from pigs and these animals might be a source of human 2/O:3 infections [2].

Wild animals constitute a very important factor in the epidemiology of *Yersinia* infection [3, 4], and wild boars (*Sus scrofa*) are considered an important reservoir of enteropathogenic *Yersinia* [5]. A great variety of serotypes, including those that cause human infections, have been isolated from wild boars in Europe [3, 5, 6], although some *Y. enterocolitica* strains differ from those in domestic pigs [2].

More studies are required to understand the real role of wild boars in the epidemiology of yersiniosis. During the last two decades, the wild boar population has increased significantly in Europe [7], favouring their contact with livestock and the transmission of diseases [8]. Interest in wild boars as a meat source has also increased, thus increasing the risk of the transmission of food-borne diseases [9].

The prevalence of pathogenic* Yersinia* spp. in Spanish wild boars is unknown. Therefore, the aim of this study was to determine the prevalence of* Y. enterocolitica* and* Y. pseudotuberculosis* in wild boars in northern Spain.

## Methods

### Study area

The Basque country is located in northern Spain, limited by the Cantabrian coastline and distributed in eight regions, defined according to rainfall, temperature, altitude and the dominant vegetation [10, 11]. Climatologically, the Atlantic slope (northern part) is moderate in terms of temperature, but very rainy, whereas the Mediterranean slope (southern part) is less rainy, with warmer summers and colder winters.

### Sample collection

Wild boar samples were collected within the context of a wildlife health surveillance program in the Basque Country. In total, 505 wild boars were sampled between 2001 and 2012, during which time 490 serum samples were obtained, and in the last 3 years, 72 tonsils were also collected. Both serum and tonsil samples were obtained from only 57 animals. Most of the animals studied (90 %) had been shot by accredited hunters, and samples were taken in the field in collaboration with competent local authorities, and 8 % were obtained from wildlife rehabilitation centres. The cause of death and the health status of these animals were not recorded. The remaining samples (2 %) were obtained from animals found dead or run over, and necropsies were performed in the laboratory. No significant lesions, except physical trauma, were observed in these animals. The samples were collected in individual containers, properly identified and stored at −20 °C until analysis. The details of each animal, including its sex, age, and the date and geographic location of collection were recorded. The animals were classified into two groups according to age: young, including piglets (<1 year) and yearlings (1–2 years); and adults (>2 years). Details of the animals are given in Tables 1 and 2.

**Table 1:** Seroprevalence of pathogenic *Yersinia* spp. detected in wild boars according to the variables studied

Variables	N	ELISA (%)
Age		
Young	102	42 (41.2)
Adult	98	81 (82.7)
Sex		
Females	104	66 (63.5)
Males	118	72 (61)
Sampling year		
2001	12	7 (58.3)
2002	10	10 (100)
2003	167	74 (44.3)
2004	80	41 (51.3)
2005	67	47 (70.2)
2006	53	39 (73.6)
2010	25	12 (48)
2011	40	15 (37.5)
2012	17	9 (52.9)
Season		
Winter	168	108 (64.3)
Spring	29	19 (65.5)
Summer	5	0
Autumn	269	127 (47. 2)
Natural regions		
1	298	147 (49.3)
2	90	52 (57.8)
3	1	1 (100)
4	17	17 (100)
6	4	4 (100)
Slope		
Atlantic	445	217 (48.8)
Mediterranean	42	37 (88.1)
Porcine census		
Low (10–140)	81	44 (54.3)
Middle (167–426)	219	119 (54.3)
High (580–7332)	162	82 (50.6)
Caprine census		
Low (66–655)	71	53 (74.7)
Middle (909–1056)	234	111 (47.4)
High (1136–2810)	157	81 (51.6)
Ovine census		
Low (1881–6698)	102	60 (58.8)
Middle (8035–15,033)	138	91 (65.9)
High (15,417–32,802)	222	94 (42.3)
Bovine census		
Low (276–4277)	132	77 (58.3)
Middle (4602–6768)	172	103 (59.9)
High (6781–19,109)	158	65 (41.1)

*N* number of samples analyzed, *ELISA* number and percentage of ELISA positive samples

**Table 2:** Prevalence of pathogenic *Yersinia* detected with rt-PCR in wild boars according to the variables studied

Variables	N	YE and YP (%)	YE (%)	YP (%)
Age				
Young	25	18 (72)	12 (48)	9 (36)
Adult	20	10 (50)	8 (40)	3 (15)
Sex				
Females	30	19 (63.3)	12 (40)	11 (36.7)
Males	19	12 (63.2)	9 (47.4)	4 (21.1)
Sampling year				
2010	23	18 (78.3)	13 (56.5)	9 (39.1)
2011	32	7 (21.9)	7 (21.9)	0
2012	17	12 (70.6)	4 (23.5)	9 (52.9)
Season				
Winter	8	5 (62.5)	5 (62.5)	1 (12.5)
Spring	9	7 (77.8)	2 (22.2)	6 (66.7)
Summer	10	5 (50)	3 (30)	2 (20)
Autumn	45	20 (44.4)	14 (31.1)	9 (20)
Natural regions				
1	58	26 (44.8)	15 (25.9)	15 (25.9)
2	6	5 (83.3)	5 (83.3)	0
Slope				
Atlantic	72	37 (51.4)	24 (33.3)	18 (25)
Porcine census^a^				
Low	26	12 (46.1)	6 (23.1)	8 (30.8)
Middle	24	11 (45.8)	6 (25)	6 (25)
High	22	14 (63.6)	12 (54.6)	4 (18.2)
Caprine census^b^				
Low	3	0	0	0
Middle	35	19 (54.3)	10 (28.6)	12 (34.3)
High	34	18 (52.9)	14 (41.2)	6 (17.7)
Ovine census^c^				
Low	20	7 (35)	3 (15)	4 (20)
Middle	28	12 (42.9)	6 (21.4)	8 (28.6)
High	24	18 (75)	15 (62.5)	6 (25)
Bovine census^d^				
Low	26	13 (50)	6 (23.1)	9 (34.6)
Middle	17	7 (41.2)	4 (23.5)	3 (17.7)
High	29	17 (58.6)	14 (48.3)	6 (20.7)

*N* number of samples analyzed, *YE* and *YP* number and percentage of *Y. enterocolitica* and *Y. pseudotuberculosis* positive samples, *YE* number and percentage of *Y. enterocolitica* positive samples, *YP* number and percentage of *Y. pseudotuberculosis* positive samples

^a^Porcine census: low (10–140), middle (167–426), high (580–7332)

^b^Caprine census: low (66–655), middle (909–1056), high (1136–2810)

^c^Ovine census: low (1881–6698), middle (8035–15,033), high (15,417–32,802)

^d^Bovine census: low (276–4277), middle (4602–6768), high (6781–19,109)

### Real-time polymerase chain reaction

The tonsil samples (1–5 g) were weighed and aseptically cut into small pieces. Approximately 150 mg of each tonsil was disrupted and homogenised with 30 chrome–steel beads (1.3 mm) (Biospec Products, Bartlesville, OK, USA) and 750 µL of TE buffer using the TissueLyser system (Qiagen, Hilden, Germany). DNA was extracted from 200 µL of the supernatant for direct real-time polymerase chain reaction (rt-PCR) analysis. The rest of each tonsil sample was mixed with phosphate-buffered saline (PBS) supplemented with 1 % mannitol (Fluka, Seelze, Germany) and 0.15 % bile salts (Fluka, Seelze, Germany) (PBS-MSB), diluted 1:10 and homogenised in a stomacher (Lab-Blender 80, Cole-Parmer, Vernon Hills, IL, USA) until homogeneity. The mixture was incubated for 14 days at 4 °C. DNA was extracted from 200 µL of the supernatant and used as the template for rt-PCR.

DNA extraction was performed with the QIAamp^®^ DNA Blood Mini Kit (Qiagen), according to the manufacturer’s instructions, with minor modifications [12], and the DNA was measured with a NanoDrop ND-1000 spectrophotometer (Thermo Scientific, Inc.). DNA (150–200 ng) was used to detect *Yersinia* with the TaqMan rt-PCR assay in three independent reactions, using the Applied Biosystems 7500 Real-Time PCR System and The Express qPCR Supermix, universal kit (Invitrogen™), according to the supplier’s recommendations. *Yersinia enterocolitica* was detected with the amplification of the *ail* gene [13], using a previously described procedure [12]. To detect all the *Y. pseudotuberculosis* serotypes, the *wzz* and *ail* genes were amplified in two independent reactions [12, 14, 15]. Amplification of the *ail* gene detects all serotypes but O:11 and O:12, and amplification of the *wzz* gene detects all serotypes but O:6 and O:7 [14, 15]. A sample was considered positive for *Y. enterocolitica* or *Y. pseudotuberculosis* when at least one positive result was obtained in the direct reaction or after enrichment in any of the three rt-PCRs used.

### Enzyme-linked immunosorbent assay

The presence of antibodies directed against pathogenic *Yersinia* was determined with a commercial indirect enzyme-linked immunosorbent assay (ELISA) specific for swine (PIGTYPE^®^ YOPSCREEN, Labor Diagnostic, Leipzig, Germany), according to the manufacturer’s instructions. The optical density (OD) was measured in an ELISA Multiskan (Thermo Labsystem) spectrophotometer at 450 nm. The ratio between the sample OD and the positive control OD (S/P ratio) was calculated. Samples with an S/P ratio ≥0.3 were considered positive.

### Bacteriology

Selective cefsulodin–irgasan–novobiocin (CIN) agar (bioMérieux, Marcy l’Etoile, France) and CHROMagar™ *Y. enterocolitica* (CHROMagar, Paris, France) agar were inoculated with 20 µL of the rt-PCR-positive tonsil mixtures and incubated at 30 °C for 24–48 h to isolate the *Yersinia* strains. Red CIN agar “bull’s-eye” colonies surrounded with a transparent area of 1 mm and mauve CHROMagar™ colonies were selected. The selected colonies were homogenised in 500 µL of PBS, and 50 µL of this mixture was incubated for 10 min at 100 °C in a water bath and then for 10 min on ice. The mixture was then centrifuged for 10 min at 15,600×*g* and 5 µL of the supernatant was used for *Y. enterocolitica* and *Y. pseudotuberculosis* identification with rt-PCR, with the procedures described above. The colonies were also streaked directly onto triple sugar iron agar (Oxoid Ltd, Basingstoke, England) and onto blood agar (bioMérieux) and identified with the VITEK system (bioMérieux), using a previously reported protocol [12].

The *Yersinia* strains were serotyped with slide agglutination using commercial *Y. enterocolitica* O:1, O:2, O:3, O:5, O:8 and O:9 antisera (Denka Seiken, Coventry, UK), *Y. enterocolitica* O:27 antiserum (SIFIN, Berlin, Germany) and *Y. pseudotuberculosis* O:1–O:6 antisera (Denka Seiken). *Yersinia pseudotuberculosis* was also serotyped with O-genotyping, using a conventional multiplex PCR, according to Bogdanovich et al. [16].

### Data analysis

All statistical analyses were performed in the SAS 9.3 software. The official 2009 livestock census data were obtained from the Basque Statistics Institute (http://www.eustat.es) for each region and the PROC RANK Statement was used to classify each region as containing high, medium, or low numbers of each species. The relationships between *Yersinia* prevalence and the different independent variables studied (sex, age, sampling year, season, natural region, slope and livestock numbers) were examined statistically using the χ^2^ or Fisher’s test. The simple kappa coefficient of agreement was used to determine the degree of agreement between the ELISA and PCR results when applied to the same animal. A *t* test was used to compare the ELISA S/P ratios between the PCR-positive and -negative animals. Differences were considered significant at *P* < 0.05.

## Results

**Fig. 1:**
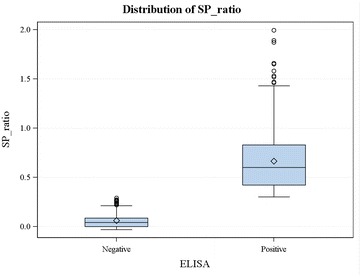
Distribution of ELISA S/P ratios for *Yersinia* spp. detected in 490 wild boars. Each of the two *box plots* represents the summary statistics for the S/P ratios of the ELISA-positive and -negative samples. *Boxes* represent the 25 and 75 % percentiles; the *horizontal lines* indicate the median values for the S/P ratios; the *diamond shapes* represent the mean S/P ratios; and the *vertical lines* extend from the minimum S/P ratios to the maximum ratios

Antibodies directed against pathogenic *Yersinia* were detected in 52.5 % (257/490) of the wild boars. The mean S/P ratio was 0.66 (95 % confidence interval [CI] 0.63–0.70) for the ELISA-positive samples and 0.061 (95 % CI 0.05–0.07) for the ELISA-negative samples (Fig. 1).

*Yersinia* infection was detected with rt-PCR in 51.4 % (37/72) of wild boars. *Yersinia enterocolitica* was present in 33.3 % (24/72) and *Y. pseudotuberculosis* in 25.0 % (18/72) of the animals. Mixed infections of *Y. enterocolitica* and *Y. pseudotuberculosis* were identified in five individuals. Ten of the 18 *Y. pseudotuberculosis*-positive samples were detected with the amplification of both the *ail* and *wzz* genes, four with the amplification of only *ail*, and the other four with the amplification of only the *wzz* gene.

Of the 37 rt-PCR-positive samples, 23 were only positive after enrichment and nine were only positive on direct rt-PCR. Eight samples were positive on both direct rt-PCR and after enrichment, but lower cycle threshold (Ct) values were obtained after enrichment (see Additional file 1).

Seroprevalence was higher in the adult animals than in the young animals (*P* < 0.0001; Table 1), but no significant differences were observed according to age with PCR (*P* = 0.2157; Table 2).

Significant differences were observed according to the sampling year. The highest seroprevalence was detected in 2002 and in 2005–2006, although in 2002, only 10 samples were analysed (*P* < 0.0001; Table 1). The prevalence of *Y. enterocolitica* was highest in 2010 (*P* = 0.0213) and that of *Y. pseudotuberculosis* was highest in 2012 (*P* < 0.0001; Table 2).

The overall seroprevalence was highest in winter and spring (*P* < 0.0001; Table 1). The prevalence of *Y. pseudotuberculosis* was highest in spring (*P* = 0.0305), but no significant difference was observed in the prevalence of *Y. enterocolitica* between seasons (*P* = 0.3180; Table 2).

**Fig. 2:**
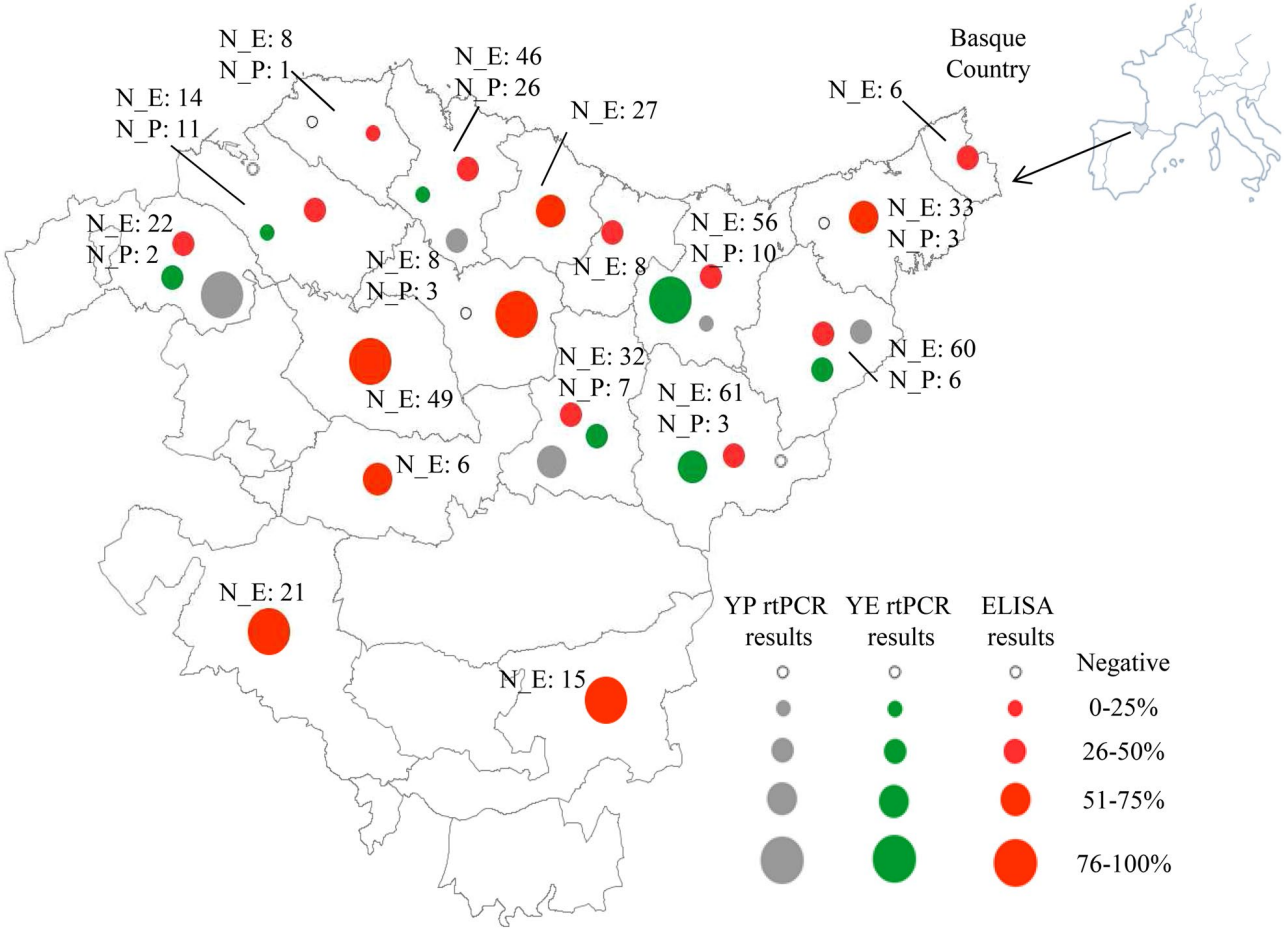
Geographic distributions of the ELISA and PCR results for *Y. enterocolitica* and *Y. pseudotuberculosis* in wild boars in northern Spain. Prevalence of pathogenic *Yersinia* spp. detected with rt-PCR and ELISA is illustrated with points of different sizes and colours. *N_P* Number of wild boars analysed with rt-PCR; *N_E* Number of wild boars analysed with ELISA

Statistically significant differences were observed in the seroprevalence of *Yersinia* spp. according to the slope and region of habitation (*P* < 0.0001; Table 1). These differences were also significant for *Y. enterocolitica* and regions (*P* = 0.0096; Table 2). The geographic distribution of the positive samples is illustrated in Fig. 2.

Higher seroprevalence was observed in areas with small livestock populations (caprine, *P* < 0.0001) or medium livestock populations (bovine, *P* = 0.0011; ovine, *P* < 0.0001) (Table 1), whereas *Y. enterocolitica* prevalence was highest in areas with large ovine populations (*P* = 0.0012; Table 2).

Two isolates of *Y. pseudotuberculosis* and two of *Y. enterocolitica* were collected from four different wild boars. The *Y. pseudotuberculosis* isolates were obtained on CIN agar, one with direct plating and the other after enrichment. Both *Y. enterocolitica* isolates were obtained after enrichment, one on CIN agar and the other on CHROMagar™. The identities of *Y. enterocolitica* and *Y. pseudotuberculosis* were confirmed for each isolate with rt-PCR amplification of the *ail* gene. No agglutination was detected when the *Y. pseudotuberculosis* isolates were serotyped with the antisera used, but both isolates were identified as serotype O:1c with multiplex O-gene amplification. It was not possible to serotype the *Y. enterocolitica* isolates because of contamination.

Of the 57 wild boars analysed with rt-PCR and ELISA, 13 were positive and 19 were negative with both techniques, seven animals were positive only according to ELISA, and 18 animals were positive only according to rt-PCR (κ index = 0.1452). No differences were observed in the ELISA S/P ratios when the *Y. enterocolitica*-rt-PCR-positive and -negative animals were compared. However, the *Y. pseudotuberculosis*-positive animals had higher S/P ratios (mean 0.53; 95 % CI 0.21–0.86) than the *Y. pseudotuberculosis*-negative animals (mean 0.23; 95 % CI 0.12–0.35; *P* = 0.0249).

## Discussion

This study demonstrates that *Y. enterocolitica* and *Y. pseudotuberculosis* infections are widespread among the wild boars in northern Spain. The seroprevalence was high (52.5 %), although slightly lower than those detected in wild boars in Germany and Switzerland (62.6 and 65.0 %, respectively) [5, 17]. The prevalence of *Y. enterocolitica* and *Y. pseudotuberculosis* can also be considered high (33.5 and 25 %, respectively) because their observed prevalence in wild boars in Europe ranges from 4.35 to 35 % for *Y. enterocolitica* and is around 20 % for *Y. pseudotuberculosis* [5, 18, 19]. *Yersinia enterocolitica* was more prevalent than *Y. pseudotuberculosis*, as is usually found in wild boars and pigs [5, 20]. Mixed infections were detected in a proportion of the animals, as previously described [5], but the prevalence of *Y. pseudotuberculosis* (25 %) was higher than expected in wild boars or organically produced pigs, probably because they are in frequent contact with other infected wild species and livestock in extensive grazing systems [4, 21]. The use of two different rt-PCR methods and the higher detection rates recorded when an enrichment step was included before rt-PCR, could also have improved the detection rate for *Y. pseudotuberculosis* [12].

The highest seroprevalence was detected in spring and winter, which is attributable to the highest *Y. pseudotuberculosis* prevalence recorded in spring and the (not significantly) highest *Y. enterocolitica* prevalence recorded in winter. To the best of our knowledge, the seasonality of *Y. enterocolitica* and *Y. pseudotuberculosis* infections has not been reported previously in wild boars. However, in other wildlife species, the disease is usually detected in the coldest months of the year [22] or from November to May, which is related to the birth of newborns [23].

The highest seroprevalence and presence of *Y. enterocolitica* were associated with mountainous areas at altitudes higher than 600 m, very cold winters, and the highest annual rainfall for each dominant climate. A similar trend was observed in pigs slaughtered in China, in which the incidence of *Y. enterocolitica* was higher in cold areas than in warm areas [24].

The highest prevalence of *Y. enterocolitica* was detected in areas with a high ovine presence. Sheep have been described as a reservoir of pathogenic *Y. enterocolitica* and *Y. pseudotuberculosis* [25, 26], but little is known about the infection of sheep in Spain with pathogenic *Yersinia* or their relationship with the *Yersinia* species found in wild boars, although *Yersinia* is reported to cause sporadic abortion in sheep in the area studied [27]. In contrast, the highest *Yersinia* seroprevalence was associated with medium or low numbers of other livestock, suggesting that other wildlife species also contribute to the epidemiology of *Yersinia* infection among wild boars. However, more studies are required to determine the real impact of pathogenic *Yersinia* on livestock in this area.

The rates of isolation were low, despite the use of two different culture media, including CHROMagar™, which is recommended for the isolation of *Y. enterocolitica* [28]. *Y. enterocolitica* pathogenicity therefore remains unknown, because the *ail* gene is an insufficient marker of virulence, and is also present in some *Y. enterocolitica* biotype 1A strains [29]. The only two *Y. pseudotuberculosis* strains isolated were identified as serotype O:1c. Little is known about the infection of animals or humans by serotype O:1c because the majority of studies have not included this subserotype. However, *Y. pseudotuberculosis* serotype O:1 has been described as one of the most commonly found serotypes infecting wild boars, pigs and humans in Europe [2, 30, 31]. This fact highlights the need for the better characterisation of its pathogenicity.

More efforts are required to isolate and characterise the *Yersinia* strains from infected wild boars in the Basque country to determine their pathogenicity and any potential risk they pose to humans and domestic species.

## Conclusions

This study demonstrates that *Y. enterocolitica* and *Y. pseudotuberculosis* are highly prevalent among wild boars in the Basque Country, with *Y. enterocolitica* the most frequently found species. The risk of infection among wild boars is influenced by the season and the area in which the animals live.

## Additional file

**Additional file 1.** ELISA and rt-PCR results for 72 wild boars in which tonsils were available. Detailed information on the rt-PCR results and cycle threshold values for samples obtained from 72 wild boars in which tonsils were available. The ELISA results and S/P ratios obtained from the animals are also included.
