# Supplementary material for: Prevalence of Yersinia enterocolitica and Yersinia pseudotuberculosis in wild boars in the Basque Country, northern Spain
Source: Acta Vet Scand. 2016 Jan 20;58:4. doi: 10.1186/s13028-016-0184-9 (PMC4719535; doi:10.1186/s13028-016-0184-9)
Supplement: Supplementary file 1 — Additional file 1. ELISA and rt-PCR results for 72 wild boars in which tonsils were available. Detailed information on the rt-PCR results and cycle threshold values for samples obtained from 72 wild boars in which tonsils were available. The ELISA results and S/P ratios obtained from the animals are also included. [file 13028_2016_184_MOESM1_ESM.pdf]

Additional file 1. ELISA and rtPCR results obtained from 72 wild boars in which tonsils were available.

		YE *ail* gen (Ct value)		YP *ail* gen (Ct value)		YP *wzz* gen (Ct value)	
Animal ID	ELISA (S/P ratio value)	Direct rtPCR	rtPCR after enrichment	Direct rtPCR	rtPCR after enrichment	Direct rtPCR	rtPCR after enrichment
1	Pos. (0.33)	Neg.	Pos. (32.1)	Neg.	Neg.	Neg.	Neg.
2	Pos. (0.84)	Neg.	Pos. (32.7)	Pos. (39.7)	Pos. (27.0)	Pos. (36.9)	Pos. (27.8)
3	Pos. (0.77)	Neg.	Pos. (35.1)	Neg.	Neg.	Neg.	Neg.
4	Pos. (0.38)	Pos. (35)	Pos. (28.6)	Neg.	Neg.	Neg.	Neg.
5	Pos (0.41)	Neg.	Neg.	Pos. (38.8)	Neg.	Pos. (36.0)	Neg.
6	Neg. (0.06)	Neg.	Neg.	Neg.	Neg.	Pos. (38.3)	Neg.
7	Neg. (0.04)	Neg.	Pos. (27.2)	Neg.	Neg.	Neg.	Neg.
8	Neg. (0.09)	Neg.	Pos. (33.2)	Neg.	Neg.	Neg.	Neg.
9	Neg. (0.21)	Neg.	Pos. (33.1)	Neg.	Neg.	Neg.	Neg.
10	Neg (0.00)	Neg.	Pos. (30.5)	Neg.	Neg.	Neg.	Neg.
11	Neg. (0.00)	Neg.	Pos. (27.6)	Neg.	Neg.	Neg.	Neg.
12	Pos. (0.81)	Neg.	Neg.	Pos. (37.5)	Neg.	Pos. (43.1)	Neg.

13	Pos. (0.37)	Pos. (36.9)	Pos. (23.8)	Neg.	Pos. (37.1)	Neg.	Pos.(25.7)
14	Neg. (0.04)	Neg.	Pos. (23.8)	Neg.	Neg.	Neg.	Neg.
15	Neg. (0.06)	Neg.	Pos. (33.1)	Neg.	Neg.	Neg.	Neg.
16	Neg. (0.18)	Neg.	Pos. (34.7)	Neg.	Neg.	Neg.	Neg.
17	NA	Neg.	Pos. (21.1)	Neg.	Neg.	Neg.	Neg.
18	Neg. (0.05)	Pos. (24.8)	Neg.	Neg.	Neg.	Neg.	Neg.
19	Neg. (0.07)	Pos. (41.3)	Neg.	Neg.	Neg.	Neg.	Neg.
20	Neg (0.22)	Pos. (27.7)	Pos. (22.1)	Neg.	Neg.	Neg.	Neg.
21	Neg. (0.04)	Pos. (33.7)	Pos. (27.9)	Neg.	Neg.	Pos. (23.6)	Pos. (18.8)
22	Pos. (1.12)	Pos. (35.1)	Pos. (32.0)	Neg.	Neg.	Pos. (29.1)	Pos. (28.2)
23	Pos. (0.50)	Pos. (33.0)	Pos. (26.5)	Neg.	Neg.	Neg.	Neg.
24	Neg. (0.01)	Neg.	Neg.	Pos. (37.8)	Neg.	Neg.	Neg.
25	Neg. (0.05)	Neg.	Neg.	Neg.	Neg.	Pos. (36.5)	Neg.
26	NA	Neg.	Neg.	Neg.	Pos. (17.5)	Neg.	Pos. (16.0)
27	Neg. (0.03)	Neg.	Neg.	Neg.	Pos. (27.6)	Neg.	Pos. (26.9)
28	Pos. (1.02)	Neg.	Pos. (34.7)	Neg.	Neg.	Neg.	Neg.

29	Neg. (0.03)	Neg.	Pos. (33.7)	Pos. (29.1)	Pos. (19.3)	Pos. (29.7)	Pos. (17.9)
30	Pos. (0.99)	Neg.	Neg.	Neg.	Pos. (20.3)	Neg.	Pos. (17.9)
31	Neg. (0.07)	Neg.	Neg.	Neg.	Pos. (25.3)	Neg.	Pos. (24.4)
32	Pos. (1.66)	Neg.	Neg.	Pos. (35.9)	Neg.	Neg.	Neg.
33	Pos. (1.53)	Neg.	Neg.	Pos.(33.6)	Neg.	Pos. (35.2)	Neg.
34	NA	Neg.	Neg.	Neg.	Pos. (39.9)	Neg.	Neg.
35	NA	Neg.	Pos. (24.4)	Neg.	Neg.	Neg.	Neg.
36	NA	Neg.	Neg.	Neg.	Pos. (38.3)	Neg.	Neg.
37	NA	Neg.	Pos. (31.2)	Neg.	Neg.	Neg.	Neg.
38	Pos (0.44)	Neg.	Neg.	Neg.	Neg.	Neg.	Neg.
39	Pos (0.53)	Neg.	Neg.	Neg.	Neg.	Neg.	Neg.
40	Pos (0.38)	Neg.	Neg.	Neg.	Neg.	Neg.	Neg.
41	Pos (0.68)	Neg.	Neg.	Neg.	Neg.	Neg.	Neg.
42	Pos (0.36)	Neg.	Neg.	Neg.	Neg.	Neg.	Neg.
43	Pos. (1.99)	Neg.	Neg.	Neg.	Neg.	Neg.	Neg.
44	Pos. (0.31)	Neg.	Neg.	Neg.	Neg.	Neg.	Neg.

45-63	Neg.	Neg.	Neg.	Neg.	Neg.	Neg.	Neg.
64-72	NA	Neg.	Neg.	Neg.	Neg.	Neg.	Neg.

YE: Y. enterocolitica

YP: *Y. pseudotuberculosis*

Neg.: Negative

Pos.: Positive

NA: Serum sample was not available
